# Novel pathogenic variant in LMNA gene identified in a six-generation family causing atrial cardiomyopathy and associated right atrial conduction arrhythmias

**DOI:** 10.3389/fcvm.2023.1109008

**Published:** 2023-07-03

**Authors:** Shifeng Ning, Min Han, Rujie Qiu, Xiaoming Hong, Zhao Xia, Li Liu, Chengwei Liu

**Affiliations:** Division of Cardiac Care Unit, Department of Cardiology, Wuhan Asia Heart Hospital, Wuhan, China

**Keywords:** *LMNA* gene mutation, pathogenic variant, atrial cardiomyopathy, right atrial enlargement, atrial standstill, bicuspid aortic valve malformation

## Abstract

**Objective:**

To characterize the cardiac phenotype associated with the novel pathogenic variant (c.1526del) of *LMNA* gene, which we identified in a large, six-generation family.

**Methods and Results:**

A family tree was constructed. The clinical data of living and deceased family members were collected. DNA samples from 7 family members were analyzed for *LMNA* mutations using whole-exome high-throughput sequencing technology. The clinical presentation of pathogenic variant carriers was evaluated. In this six-generation family (*n* = 67), one member experienced sudden death at the age of 40-years-old. Three pathogenic variant carriers were identified to possess a novel heterozygous deletion mutation in *LMNA* gene (HGVS: NM_170707.4, c.1526del) located at exon 9 of *LMNA* chr1:156137145, which creates a premature translational stop signal (p.Pro509Leufs*39) in the LMNA gene and results in an mutant lamin A protein product. The main symptoms of the pathogenic variant carriers were palpitation, fatigue, and syncope, which typically occurred around 20-years-old. AV-conduction block and non-sustained ventricular tachycardia were the first signs of disease and would rapidly progress to atrial standstill around 30-years-old. Significant right atrial enlargement and bicuspid aortic valve malformation was also commonly seen in patients who carried this pathogenic variant.

**Conclusion:**

The pathogenic variant of c.1526del p.P509Lfs*39 was a frameshift deletion located at exon 9 of *LMNA* chr1:156137145 and causes severe right atrial enlargement, sick sinus syndrome, atrial standstill, ventricular tachycardia, and bicuspid aortic valve malformation. Our findings expand the phenotypic spectrum of novel *LMNA* gene mutations.

## Introduction

The concept of atrial cardiomyopathy (ACM) was initially proposed a decade ago (1) and was widely accepted until recently. According to *EHRA/HRS/APHRS/SOLAECE expert consensus on atrial cardiomyopathies*, ACM is defined as “any complex of structural, architectural, contractile or electrophysiological changes affecting the atria with the potential to produce clinically-relevant manifestations”. It's classified into four classes: principally cardiomyocyte changes, principally fibrotic changes, combined cardiomyocyte-pathology/fibrosis, and primarily non-collagen infiltration (with or without cardiomyocyte changes) (2). However, this histological-based classification system has limited practical application for most patients due to the need for an invasive atrial biopsy to confirm diagnosis. ACM is characterized by a dilated atrium with various arrhythmias (mainly atrial arrhythmias), and was found to be associated with Ebstein anomaly (3), dilated cardiomyopathy (DCM) (4), myocarditis (5), amyloidosis (6) and muscular dystrophies (7). Although its prevalence is unknown, ACM was often observed in various clinical conditions, such as isolated atrial fibrillation, diabetes, aging, smoking, heart failure, valvular disease, amyloidosis and inflammatory infiltration, etc (2, 6). The ACM could be sporadic or familial, suggesting the possibility of genetic determinants in some forms of ACM. The first case of familial atrial cardiomyopathy was described by Nagle RE et al. in 1972 (8), who described a familial syndrome almost exclusively affecting the atria and atrioventricular-conducting system, with a combination of ectopic supraventricular rhythms and atrioventricular block that eventually progressed to atrial standstill. Thereafter, additional cases of ACM with hereditary components were reported and a variety of gene mutations associated with ACM were identified, including heterozygous mutations of *SCN5A* (9) and *GJA5* (encoding connexin 40) genes (10), the gain-of-function *KCNQ1* missense variant (11), homozygous mutation in the natriuretic peptide precursor A (*NPPA*) gene (12), nonsense mutation in the *STA* Gene (13), nonsense mutation in the *LMNA* gene (c.475G > T) (14), missense mutation in the *LMNA* gene (p.R335W) (15), etc. Currently, the *LMNA* gene, located on chromosome 1q21.1–21.2, is thought to be one of the most common disease-associated genes for familial DCM with respects to conduction system diseases (15). As of now, a total of 498 *LMNA* pathogenic variants have been identified and are associated with more than 15 different phenotypes (16, 17). Here we report a novel pathogenic variant of *LMNA* gene, discovered to be a frame-shift mutation of the *LMNA* gene at c.1526del, in a large six-generation family. The clinical manifestation was ACM, including giant right atrium, sick sinus syndrome, atrial standstill, left ventricular tachycardia, and bicuspid aortic valve malformation. To the best of our knowledge, this is a novel identification of a pathogenic variant of *LMNA* gene. Our findings facilitate improved understanding of the *LMNA* mutation genotype and its clinical phenotype to other known laminopathies.

## Materials and methods

### Ethics statement

This study was approved by the Institutional Ethical Committee on Human Research at the Wuhan Asia Heart Hospital. All patients gave written informed consent for participation in the study, including blood sampling, isolation of DNA, molecular genetic testing, and provision of their clinical data.

### Study subjects and clinical data

The pedigree was created based on information provided by the family members and their medical records. Clinical data from the proband and other living mutation-carriers were collected from their respective medical records. In addition, medical reports and examination results (electrocardiogram (ECG), Holter-ECG, pacemaker/implantable cardioverter-defibrillator (ICD) interrogations, echocardiography) of both living and deceased family members were collected if available.

### Genetic testing and sequencing data analysis

After obtaining the written informed consent, peripheral blood samples were collected from the proband and some members of the 3rd–6th generations (*n* = 24). The genomic DNA was extracted using the TIANamp Blood DNA Kit (TIANGEN, Beijing, China) according to the manufacturer's standard protocol. Genetic testing was performed by the Beijing Berry Hekang Medical Laboratory using high-throughput whole-exome sequencing technology and the Verita Trekker variant detection system. The sequencing results were analyzed using the Enliven variant site annotation interpretation system, which had been independently developed by Berry Gene Data.

## Results

### Clinical presentation of proband (V:1)

**Figure 1:**
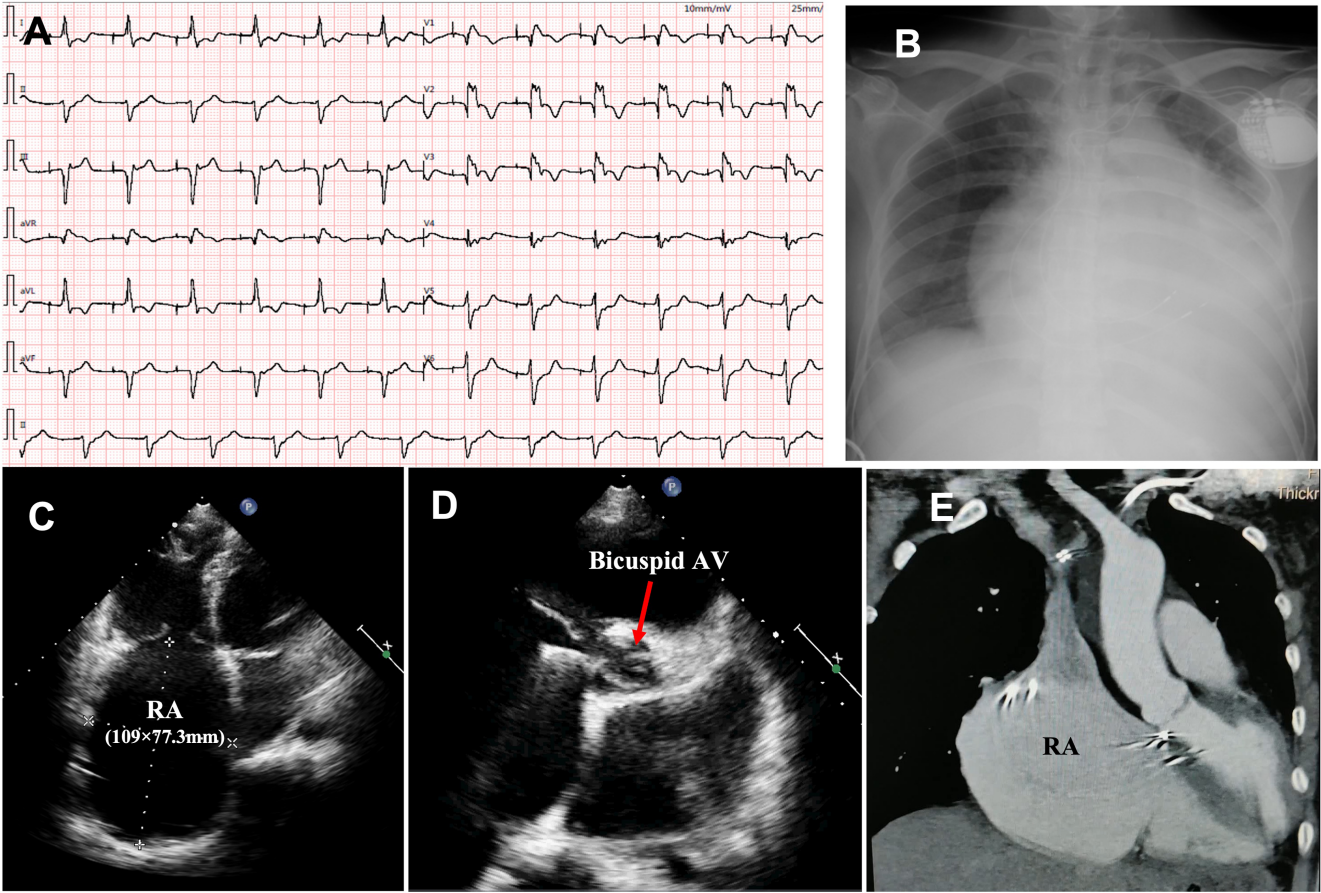
Upon admission, ECG showed pacing rhythm at 71 beats/min) (**A**); chest x-ray showed cardiac enlargement (**B**); TTE revealed an extremely enlarged RA (**C**) and bicuspid aortic valve malformation (**D**); the enlarged RA was confirmed by CTA (**E**). ECG, electrocardiogram; CXR, chest x-ray; TTE, transthoracic echocardiography; CTA, computed tomography angiography; RA, right atrium; AV, aortic valve.

**Figure 2:**
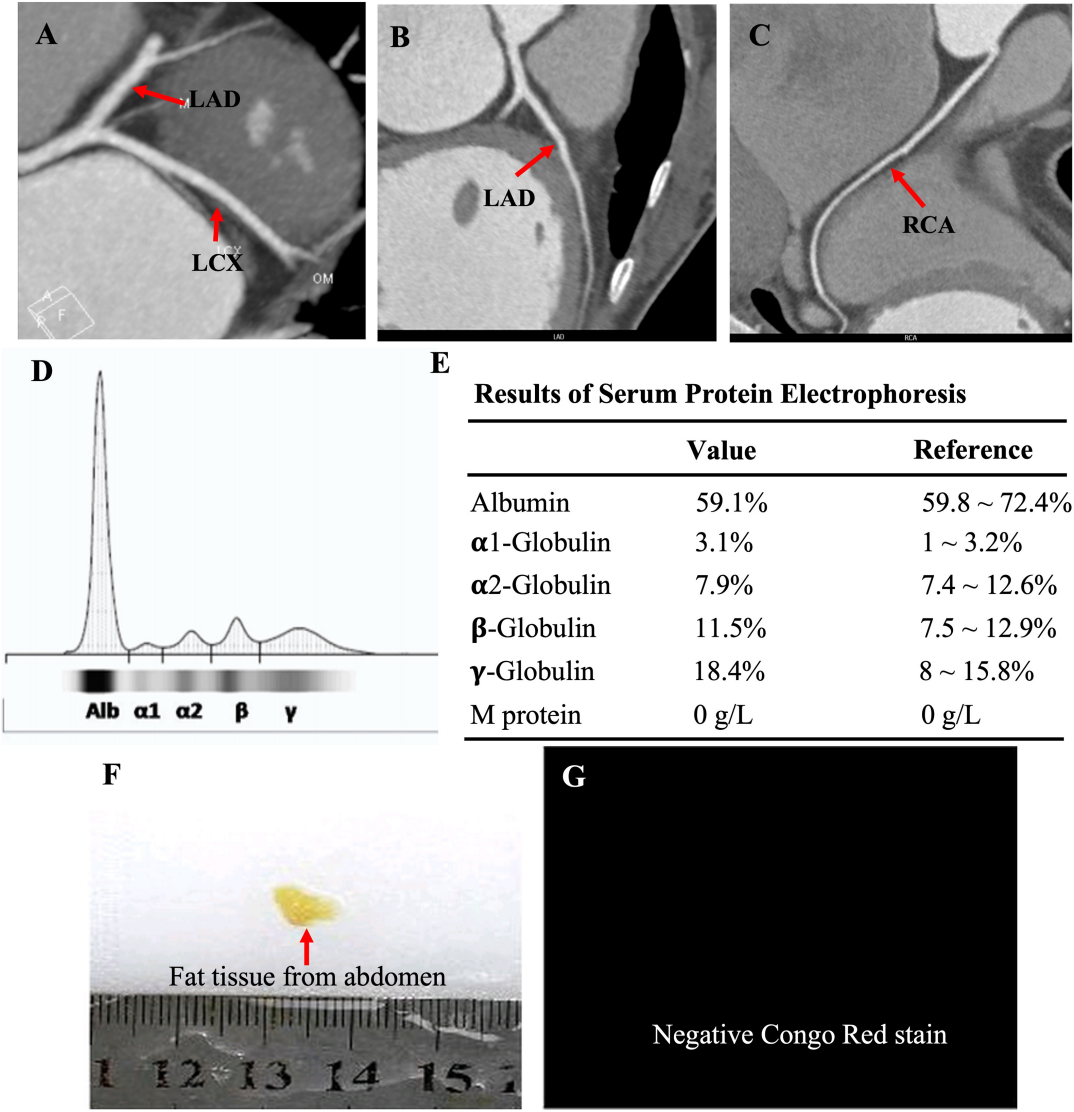
CTA showed normal coronary arteries, including the LAD, LCX and RCA (**A–C**); protein electrophoresis showed normal range of α1-, α2-, β-, γ-globulin and M-protein (**D,E**); biopsy of abdominal fat showed a negative Congo Red stain (**F,G**), ruling out potential amyloid cardiomyopathy.

**Figure 3:**
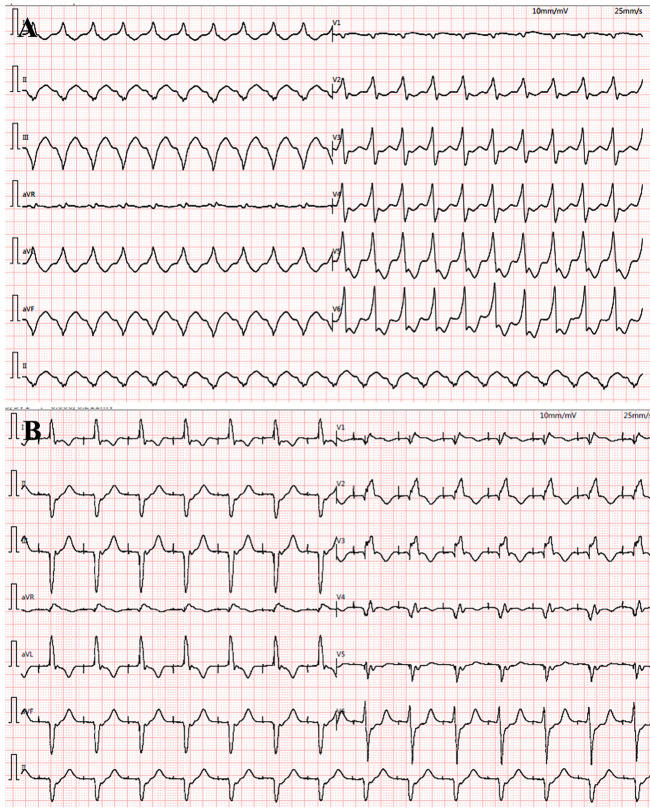
During hospitalization, ventricular tachycardia (VT) reoccurred (**A**) and was cardioverted with intravenous amiodarone treatment (**B**).

The proband was a 41-year-old woman who was born to a consanguineous family and presented to our hospital with the chief complaints of worsening palpitation and syncope for 2 weeks. A diagnosis of non-sustained ventricular tachycardia (VT) was made at a local hospital, where she was successfully cardioverted with intravenous amiodarone. She also complained of worsening dyspnea during physical activity, fatigue, and intermittent edema of both lower extremities for the past 13 years. She denied paroxysmal nocturnal dyspnea and a history of hypertension or diabetes. Past medical history included pacemaker implantation (Medtronic Sensia SEDRL1) 5 years ago due to a slow heart rate (3rd-degree AV conduction block according to medical records). Per medical records regarding the permanent pacemaker implantation, 3,830 electrodes placed either in the right atrial septum, the lateral wall or near the coronary sinus orifice could not be captured by 10 V pacing, suggesting possible atrial standstill. She also had noticeable right atrial enlargement and severe tricuspid regurgitation (TR). An ECG 8 years ago revealed a slow heart rate (40 bpm) with a junctional escape rhythm. Family history is remarkable for paternal sudden cardiac death (SCD) at the age of 40 and a sister who underwent pacemaker implantation due to sick sinus syndrome at 32-years-old at a local hospital. The proband's cousin is noted to have a very similar, although unspecified, heart disease diagnosed at the age of 40 and pacemaker implantation. Upon admission, physical examination revealed blood pressure (112/58mmHg), heart rate (71 beats/min), respiratory rate (18/min) and oxygen saturation level (99% at room air) were all within normal limits. Auscultation of the heart revealed a grade 3–4/6 systolic murmur heard in the subxiphoid area. The ECG showed a pacing rhythm of 711beats/min (Figure 1A). Cardiac enlargement, particularly right atrial dilation was seen on chest x-Ray (CXR) (Figure 1B). Transthoracic echocardiography (TTE) revealed extreme enlargement of the right atrium (RA, 109 × 77.3 mm), an enlarged right ventricular (RV) chamber (42 mm), severe TR, mild enlargement of left atrium (LA, 45 mm), left ventricular (LV) chamber (53 mm), and mild mitral regurgitation (MR) (Figure 1C). The left ventricular systolic function (LVEF) was within normal range (54%). A bicuspid aortic valve malformation (type 1A) was noticed on TTE examination without significant stenosis (forward flow velocity 1.5 m/s) (Figure 1D). The extremely large RA was confirmed via computed tomography angiography (CTA) (Figure 1E). Labwork indicated CBC, plasma electrolytes, troponin levels, several inflammatory and tumor markers, and renal function were within normal range, but slightly elevated N-terminal B-type natriuretic peptide (NT-pro-BNP, 417.7 pg/ml) and hepatic dysfunction. Coronary heart disease was ruled out by CTA (Figures 2A–C), and amyloidosis cardiomyopathy was excluded via protein electrophoresis (Figures 2D,E) and biopsy of abdominal fat (Congo Red stain) (Figures 2F,G). During hospitalization, the patient experienced a recurrent episode of syncope and ECG confirmed the diagnosis of VT responsive to intravenous amiodarone (Figures 3A,B). Given the patient's family history and suspicion of inherited cardiomyopathy, genetic testing of the patient and select family members was subsequently ordered.

### Results of genetic testing

**Figure 4:**
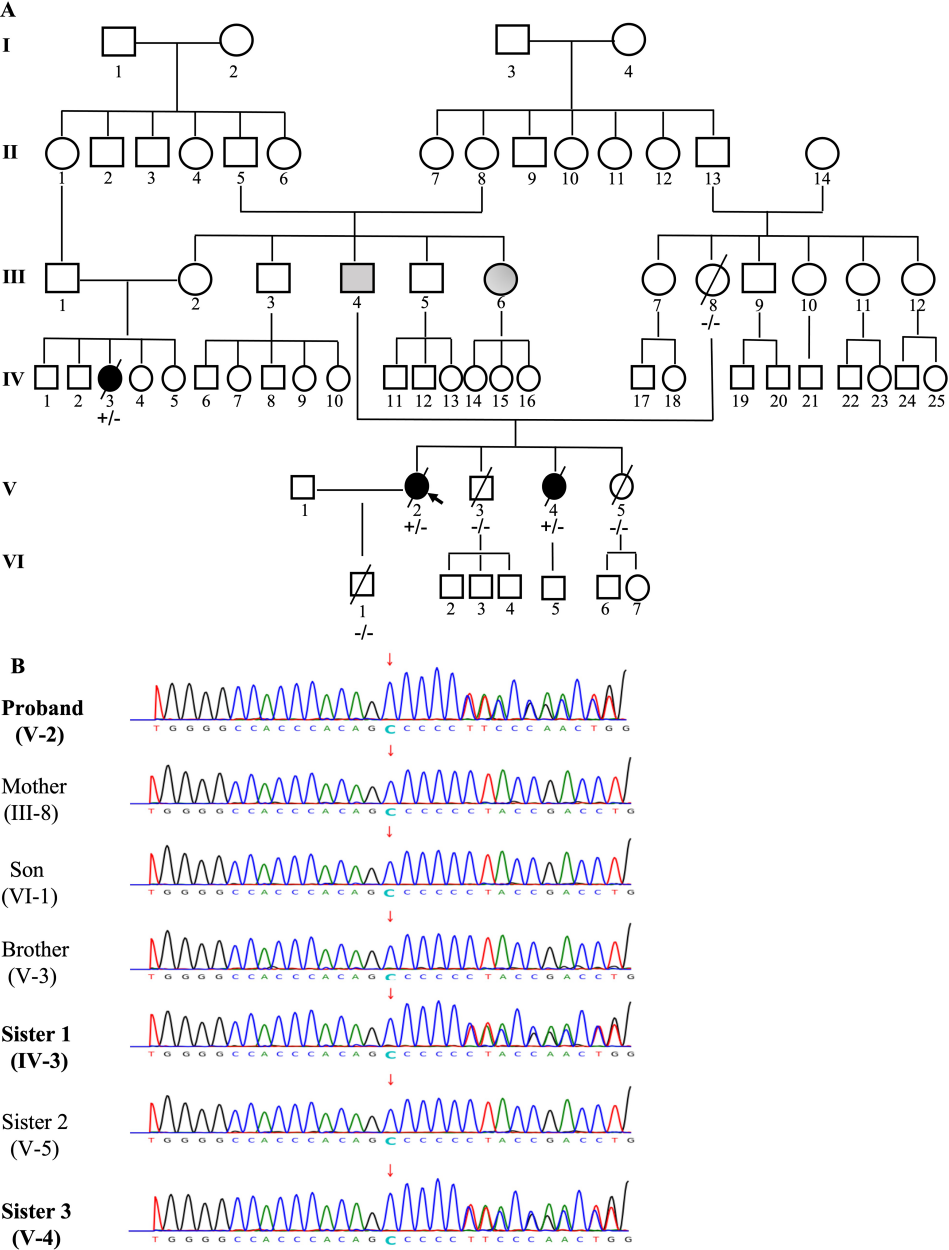
The pedigree of the family (**A**) and genetic testing results (**B**). The pedigree of this family was constructed based on a questionnaire on health history, medical records, and/or blood samples of available members that included 67 family members over six generations. Individuals are indicated by generation and pedigree number. Males and females are shown with squares and circles, respectively. Black circles/squares are affected (3 members), gray is presumedly affected (2 members), white is unaffected. Arrow indicates the proband (**A**). Genetic testing was performed in 7 members of the family (III-8, IV-3, V-2, V-3, V-4, V-5, VI-1). A pathogenic variant c.1526del: p.P509Lfs*39, a frameshift deletion in the LMNA gene in exon 9 on chromosome 1 was identified in 3 members ( IV-3, V-2, V-4), which causes changes in the open reading frame of the gene and results in loss-of-function of Lamin A/C (**B**).

The constructed family tree included 67 family members spanning over six generations (Figure 4A). Since medical records and samples were unavailable for the 1st and 2nd generations, blood or other genetic samples were only acquired from some members of the 3rd–6th generations. Although genetic samples were obtained from 24 family members, only 7 (III-8, IV-3, V-2, V-3, V-4, V-5, VI-1) eventually completed genetic testing due to cost and other reasons. In total, 1 deceased (III-4) and 1 very sick due to “heart disease” (III-6) at an early age and presumed mutation-carriers, as well as 3 genetically verified mutation-carriers (IV-3, V-2, V-4) were identified (Figure 4B). Genetic testing revealed that the proband carried a frameshift deletion in the *LMNA* gene at c.1526del (p.P509Lfs*39) in exon 9 on chromosome 1 (HGVS: NM_170707.4, c.1526del: p.P509Lfs*39), which caused changes in the open reading frame of the gene and resulted in loss-of-function of lamin A/C. This variant was not found in the China Genome Database (https://db.cngb.org), Human Exon Database (ExAC, http://exac.broadinstitute.org), Reference Population Thousand Genomes (1000G) (http://browser.1000genomes.org) or Population Genome Mutation Frequency Database (gnomAD, https://gnomad.broadinstitute.org). According to the American Society for Medical Genetics and Genomics (ACMG) guidelines (18) and Recommendations from the working group of Clingen Sequence Variant Interpretation (SVI) on the application of the guideline standards (19, 20), this c.1526del mutation of *LMNA* gene was a novel pathogenic variant with strong evidence for pathogenicity (PVS1 + PM2). Among the 7 family members who underwent genetic testing, two of the proband's sisters were found to be carrying the same pathogenic variant.

### Clinical characteristics of family members with c.1526del: p.P509Lfs*39 pathogenic variant

The patients carrying the c.1526del pathogenic variant shared some similar clinical characteristics, including clinical symptoms, ECG and TTE findings. ECG revealed a slow arrhythmia, AV-conduction block rapidly progressing to atrial standstill, and non-sustained ventricular tachycardia. TTE showed right-sided cardiac enlargement and extreme enlargement of the right atrium. Left side of the heart was less involved. A bicuspid aortic valve malformation (type A) was also typically noted on TTE examination (Table 1).

**Table 1:** Clinical characteristics of the patients carrying pathogenic variant of c.1526del: p.P509Lfs*39.

Member	IV-3	V-1 (proband)	V-3
Gender	Female	Female	Female
Onset age (years)	∼20	∼18	∼20
Clinical symptoms (initial cause of presentation)			
Palpitation	Yes	Yes	Yes
Dyspnea	Yes	Yes	Yes
Fatigue	Yes	Yes	Yes
Syncope	No	Yes	Yes
ECG findings			
AV-block	Yes	Yes	Yes
Atrial standstill	No	Yes	Yes
Non-sustained VT	Yes	Yes	Yes
Pacemaker implantation	Yes	Yes	Yes
TTE findings			
LVEF	Normal range	Normal range	Normal range
Cardiac enlargement of right-side	Yes	Yes	Yes
Cardiac enlargement of left-side	No	No	No
Severe TR	Yes	Yes	Yes
Bicuspid aortic valve malformation	?	Yes	Yes

ECG, electrocardiogram; AV-block, atrioventricular block; VT, ventricular tachycardia; TTE, transthoracic echocardiography; LVEF, left ventricular ejection fraction; TR, tricuspid regurgitation.

## Discussion

DCM is a frequent cause of heart failure and sudden cardiac death worldwide. It is now well established the significance of genetic etiology with currently more than 40 DCM genes implicated. Among them mutations on *TTN* gene (encoding titin which provides structure, flexibility, and stability to sarcomeres) was the largest contributor, following by *LMNA* gene, *SCN5A* and *DES* gene (21, 22). The *LMNA* gene is composed of 12 exons and encodes intermediate filament proteins lamin A and C via alternative splicing of exon 10 (23, 24). The lamin A/C proteins polymerize to form a scaffold called the nuclear lamina (NL) at the nuclear periphery, which consists of a long *α*-helical domain flanked by globular amino-terminal (head) and carboxy-terminal (tail) domains. Lamins first dimerize using their *α*-helical rod domain, then polymerize in a polar head-to-tail manner, forming protofilaments. Between three and four protofilaments associate laterally to form an intermediate filament (25, 26). Its expression pattern is not solely confined to the atria as it also exists in skeletal muscle and ventricular myocardium (23, 24). Lamin A/C possesses a key function in the stability of the nuclear envelope, chromatin organization, and DNA-repair (25). It plays a major role in transmitting mechanical signals to the nuclear core, transportation through the nuclear envelope, and regulation of transcription (27, 28). *LMNA* gene mutations typically present in an autosomal dominant manner and can result in a variety of phenotypes such as lipodystrophy, muscular disease, neuropathy, progeria, and cardiomyopathy (29, 30). The cardiac phenotype includes dilated cardiomyopathy, heart failure, progressive atrioventricular block (AVB), atrial fibrillation (AF), ventricular tachycardia and fibrillation (VT/VF), and SCD (16, 17, 25, 30). The most common manifestation of the cardiac phenotype is disturbance in the electrical conducting system presenting as atrioventricular block (AV-block), and both atrial and ventricular arrhythmias (31, 32). A meta-analysis by van Berlo JH et al. (31) showed that initial ECG findings in patients with the *LMNA* gene mutation appeared as low amplitude *P*-waves and PR-intervals, and 92% of patients had developed arrhythmias after age 30% and 28% received pacemaker therapy. The cardiac phenotype of the *LMNA* mutation can present as predominantly right-sided cardiomyopathy (15, 33). Zhang et al. (15) identified a pathogenic variant which was a missense variant in rod 2B domain (c.1003C > T p.R335W) of *LMNA* gene in a large Chinese family where affected members expressed clinical findings such as atrial enlargement, atrial arrhythmia, sick sinus syndrome and AV-block.

*LMNA*-related cardiomyopathy accounts for 5%–10% of familial DCM (31) and the clinical outcome is considerably worse for patients harboring *LMNA* mutations compared to those without (34, 35). Although the precise pathophysiological processes underlying the occurrence and progression of arrhythmia in patients carrying the pathogenic variant of *LMNA* gene remains unknown, the following mechanisms may be involved (1) decreased spontaneous action potential beat frequency, decreased pacemaker current (I-f) density, (2) prolonged action potential duration, increased L-type calcium current density, (3) delayed post-depolarizations (DADs), arrhythmias, and increased heart rate variability, (4) DADs, arrhythmias, and cessation of spontaneous discharges caused by beta-adrenergic stimulation and rapid pacing (36). Given the extremely poor prognosis of cardiomyopathy-causing in *LMNA* pathogenic variant carriers, early identification of affected family members is imperative. Genetic testing allows for both early identification and diagnosis of high-risk family members. If a pathogenic variant is identified in an asymptomatic individual, regular clinical cardiovascular screening (e.g., ECG) is recommended to detect the first signs of disease, which can then be managed through early intervention. If family members are not found to carry the mutation, they can be diagnosed as unaffected and do not require serial follow-up.

In this study, we identified a novel pathogenic variant in *LMNA* gene which has not been reported in literature thus far. This is a c.1526del (a single nucleotide deletion) located in exon 9 on chromosome 1 (HGVS: NM_170707.4, c.1526del: p.P509Lfs*39), causing a change in the open reading frame of the gene and resulting in loss-of-function of lamin A/C. The clinical manifestation of the affected members presented similarly to the pathogenic variant reported by Zhang et al. (15), but clinical symptoms of the c.1526del pathogenic variant presented earlier in life (∼20 years old). Although the patient's symptoms were nonspecific, auxiliary examination provided clues to warrant further investigation. ECG findings presented as gradual worsening of electrical conduction due to sinus bradycardia, sick sinus syndrome, AV-block, and atrial/ventricular arrhythmia progressing to eventual atrial electrical quiescence. A giant right atrium was a significant finding associated with this pathogenic variant and accompanied by RV enlargement and bicuspid aortic valve malformation. All affected members required eventual pacemaker implantation, with some possibly needing ICD implantation due to VT. Therefore, genetic screening of family members is necessary once familial inheritance is suspected as it could provide early diagnosis and treatment for the affected family members. Actually, a same site mutation of c.1526 (c.1526dup, p.Asn509fs) has been reported in ClinVar, however different from the pathogenic variant we identified which was a novel deletion mutation and presented as cardiac phenotype, this variants was seen in Charcot-Marie-Tooth disease type 2, cardiovascular phenotype was uncertain.

In summary, this study describes a novel cardiac phenotype caused by a newly identified pathogenic variant in *LMNA* gene (c.1526del p.P509Lfs*39) in a large family, where affected family members were clinically characterized by a giant right atrium, sick sinus syndrome, atrial/ventricular arrhythmia with progressive loss of atrial electric activity to atrial standstill, and potential accompanying bicuspid aortic valve malformation. This study expands the phenotype of the pathogenic variant in *LMNA* gene and also provides genetic information regarding atrial cardiomyopathy. To the best of our knowledge, this is a novel pathogenic variant first identified by our research team.

### Study limitations

This study carries several limitations. First, medical records and samples were unavailable in the 1st and 2nd generations, and information from members of the 3rd–6th generation may not be entirely accurate. Second, genetic testing was not performed on all living family members due to cost and difficulty with travel, so the actual number of affected members could not be identified. Lastly, as this novel pathogenic variant in *LMNA* gene was determined to be autosomal dominant, there is a possibility that the proband's pathogenic variant of *LMNA* gene may come from her father. Unfortunately, the proband's father passed at an early age so his gene mutation status could not be verified.

## Data Availability

The original contributions presented in the study are included in the article/Supplementary Material, further inquiries can be directed to the corresponding author’s.
